# Structural insights into the IgE mediated responses induced by the allergens Hev b 8 and Zea m 12 in their dimeric forms

**DOI:** 10.1038/srep32552

**Published:** 2016-09-02

**Authors:** Israel Mares-Mejía, Siseth Martínez-Caballero, Claudia Garay-Canales, Patricia Cano-Sánchez, Alfredo Torres-Larios, Samuel Lara-González, Enrique Ortega, Adela Rodríguez-Romero

**Affiliations:** 1 Instituto de Química, Universidad Nacional Autónoma de México, Circuito Exterior, Cd. Universitaria, Coyoacán, Ciudad de México, 04510; 2 Instituto de Investigaciones Biomédicas, Universidad Nacional Autónoma de México, Circuito Escolar, Cd. Universitaria, Coyoacán, Ciudad de México, 04510; 3 Instituto de Fisiología Celular, Universidad Nacional Autónoma de México, Circuito Exterior, Cd. Universitaria, Coyoacán, Ciudad de México, 04510; 4 Instituto Potosino de Investigación Científica y Tecnológica, Camino a la Presa San José 2055, Col. Lomas 4a. Sección, San Luis Potosí, México, 78216

## Abstract

Oligomerization of allergens plays an important role in IgE-mediated reactions, as effective crosslinking of IgE- FcεRI complexes on the cell membrane is dependent on the number of exposed B-cell epitopes in a single allergen molecule or on the occurrence of identical epitopes in a symmetrical arrangement. Few studies have attempted to experimentally demonstrate the connection between allergen dimerization and the ability to trigger allergic reactions. Here we studied plant allergenic profilins rHev b 8 (rubber tree) and rZea m 12 (maize) because they represent an important example of cross-reactivity in the latex-pollen-food syndrome. Both allergens in their monomeric and dimeric states were isolated and characterized by exclusion chromatography and mass spectrometry and were used in immunological *in vitro* experiments. Their crystal structures were solved, and for Hev b 8 a disulfide-linked homodimer was found. Comparing the structures we established that the longest loop is relevant for recognition by IgE antibodies, whereas the conserved regions are important for cross-reactivity. We produced a novel monoclonal murine IgE (mAb 2F5), specific for rHev b 8, which was useful to provide evidence that profilin dimerization considerably increases the IgE-mediated degranulation in rat basophilic leukemia cells.

It has been recognized that the study of protein oligomerization is relevant from several perspectives, since this phenomenon can regulate the function of the protein or can create higher-order structures^1^. An important implication of protein oligomerization has been acknowledged in type I hypersensitivity reactions, where the effect of allergen dimerization and their multivalent characteristics promotes its recognition by specific IgE antibodies bound to high affinity FcεRI receptors on the surface of mast cells and basophils^2^. This interaction triggers the cross-linking of FcεRI on the effector cell membranes, and a concomitant activation of biochemical pathways leads to degranulation and the release of various mediators such as histamine and lipids, which cause inflammatory reactions^3^. Oligomerization phenomena and its consequences have been explained for some allergens such as Ara h 1(cupin; vicillin-type protein)^4^, Ara h 3 (cupin; legumin-type protein)^5^, Bos d 5 (β-lactoglobulin)^6^, Bet v 1 (pathogenesis-related protein)^7^,^8^, Alt a 1 (β-barrel protein)^9^, Bla g 2 (aspartic proteinase from the cockroach)^10^, and Per a 3 (hemocyanin from the American cockroach)^11^.

Profilin is an ubiquitous cytoskeletal protein that interacts with several molecules, such as actin, proline-rich ligands, and phosphoinositides to perform several cellular functions. For the human profilin isoforms PFN1 and PFN2, oligomerization has been implicated in their regulation and binding to G-actin, where PFN1 acts as a tetramer^12^. It has also been reported that dimers of these human isoforms bind peptides derived from the vasodilator-stimulated phosphoprotein, thus affecting the regulation of G-actin polymerization^13^. These reports provide evidence that the oligomerization of profilin confers different properties by affecting its binding to different ligands. Profilin is also an important allergen present in pollen grains, food plants, and rubber tree, and it is considered a pan-allergen, because it is present in all eukaryotic cells and is therefore involved in allergic cross-reactions. It has recently been reported that in areas with high pollen exposure, profilin prevalence can reach 60% in allergic individuals^14^,^15^, and in those regions this allergen can induce severe food-allergic reactions, even at very low concentrations. Studies concerning the oligomerization of this plant allergen are scarce; however, it has been shown that two profilin isoforms (Art v 4.01 and Art v 4.02) from mugwort pollen form dimers and tetramers that are stabilized by disulfide bonds or ionic interactions, and it was postulated that the oligomerization of these molecules increased their allergenicity^16^. The same authors found, in western blot experiments, that oligomers of these profilin isoforms did not differ in their ability to bind serum IgE from allergic patients; when compared with the results obtained with the monomers; however, they proposed that oligomers would have additional epitopes, which would increase their allergenic potential.

Even though there have been some investigations describing profilin oligomers related to their function or in allergy, mainly using bioinformatics tools, the structural characterization of a dimeric form of a plant profilin allergen and the description of its ability to induce IgE mediated responses has not been experimentally demonstrated thus far. Here we isolated the monomeric and dimeric species of the recombinant profilin allergens rHev b 8 and rZea m 12 and obtained their crystallographic 3D structures. These two profilin allergens have a high sequence identity (79.4%) that is comparable with the differences that may exist between isoforms of the same organism. We described the molecular interactions at the interface of dimeric rHev b 8, which exhibited a disulfide bridge between the Cys-13 of both monomers that is located in a highly conserved region of the plant profilin family. A murine monoclonal IgE (mAb 2F5) specific for rHev b 8 was generated, which exhibited high affinity as determined by biolayer interferometry. A comparison of the mAb 2F5 interactions with both allergens in their monomeric and dimeric forms showed that it only recognized rHev b 8, whereas IgEs from sera of allergic individuals showed a similar recognition for both allergens in their monomeric form demonstrating cross-reactivity. Finally, we demonstrated that the dimeric form of rHev b 8 is more efficient than the monomeric form promoting significant degranulation in rat basophilic leukemia cells (RBL-2H3).

## Results

### Biochemical and structural characterization of rHev b 8 and rZea m 12

The production of recombinant profilins and the purification strategies implemented in this work, substitute the poly (L-proline) affinity columns that commonly use 3–6 M urea to elute the protein^17^, which could affect the refolding and crystallization of these proteins. The final yield for both allergens was approximately 40 mg/L of culture.

The rHev b 8 and rZea m 12 crystals (5 mg/mL) were obtained using the conditions described in the materials and methods section. These crystals diffracted to resolutions of 1.9 and 2.2 Å, respectively, and belonged to the P4_3_2_1_2 space group. When the protein concentration was increased to 10 mg/mL, only one crystal of rHev b 8 was obtained, which contained two molecules in the asymmetric unit, as indicated by a Matthews coefficient of 3.06 Å^3^/Da and solvent content of 59.9%. This crystal belonged to the space group P3_2_ and diffracted to a 2.8 Å resolution. Table 1 shows the data collection statistics for the three crystals.

**Figure 1:**
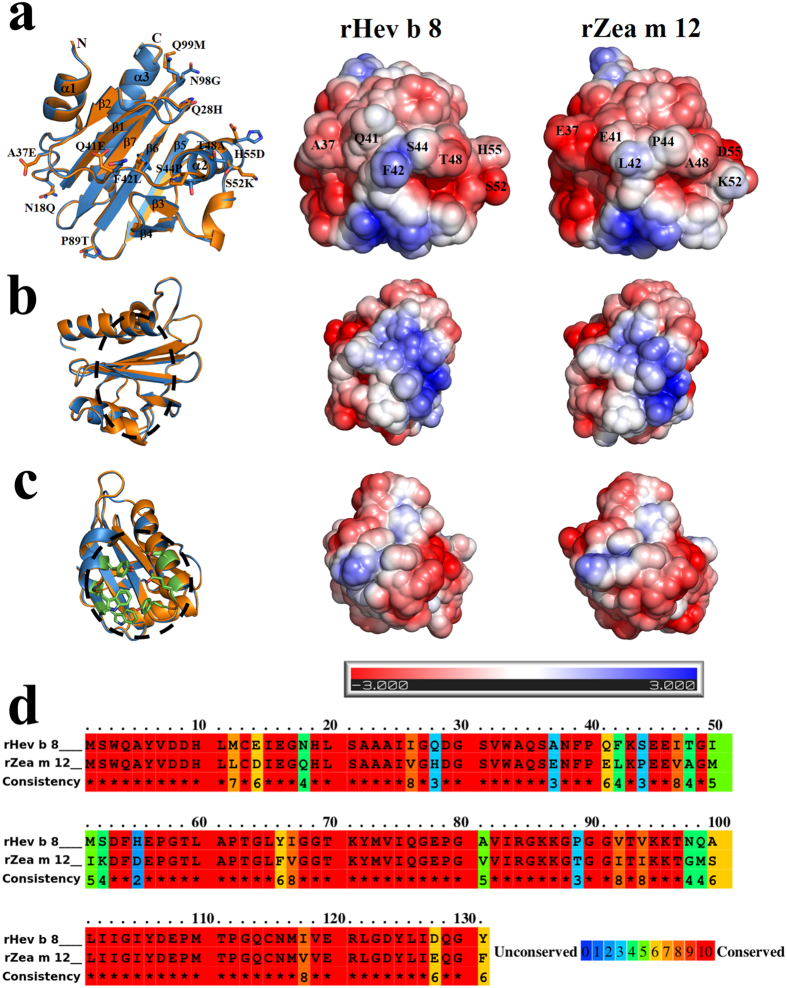
Structural comparisons of rHev b 8 and rZea m 12. (**a**) Three-dimensional alignment of rHev b 8 (blue) and rZea m 12 (orange) reveals a high structural similarity (rmsd 0.27 Å); sticks show residues that differ between the chains. The two right figures show the surface electrostatic potential of both allergens, depicting different side chains of the loop (residues Ala37 to His55 in rHev b 8 and Glu37 to Asp55 in rZea m 12). (**b**) On the left, Cα alignment of rHev b 8 and rZea m 12 showing the region of actin interaction (dashed circle); on the right, the surface electrostatic potential of both allergens in this region. (**c**) On the left, Cα alignment of rHev b 8 and rZea m 12 depicting residues involved in the poly-proline binding site (green sticks), and the comparison of the surface electrostatic potentials on the right. (**d**) Amino acid sequence alignment between rHev b 8 and rZea m 12, conserved residues are shown as a gradient from red to blue.

Overall, the rHev b 8 (PDB code 5FDS) and rZea m 12 (PDB code 5FEF) structures show the canonical fold common to all profilins, with three α helices, seven β strands in an antiparallel β-sheet, ten β-turns, and three loops. A backbone alignment of the two 3D-structures reveals that they adopt the same conformation, with a root-mean-square-deviation (rmsd) of 0.27 Å (Fig. 1a). The two conserved regions important for actin (Fig. 1b) and poly-proline binding exhibit a similar charge distribution (Fig. 1c). The sequence alignment shows that the principal differences are between residues ^**37**^**A**NFP**QF**K**S**EE**IT**G**IMS**DF**H**^**55**^for rHev b 8 and ^**37**^**E**NFP**EL**K**P**EE**VA**G**MIK**DF**D**^**55**^ for rZea m 12 (Fig. 1d). These residues are structurally located in a region exposed to the solvent, mainly in the loop that connects β-strand 2 and α-helix 2, but some also belong to α-helix 2. The electrostatic potential surfaces of both structures display the most significant differences (Fig. 1a) that lead to a more electronegative surface on rZea m 12. In addition, slight differences occur between the two proteins in some of the residues exposed to the solvent, mostly in the β-turns that connect β-strands 5 with 6 and 6 with 7 (Fig. 1a).

**Figure 2:**
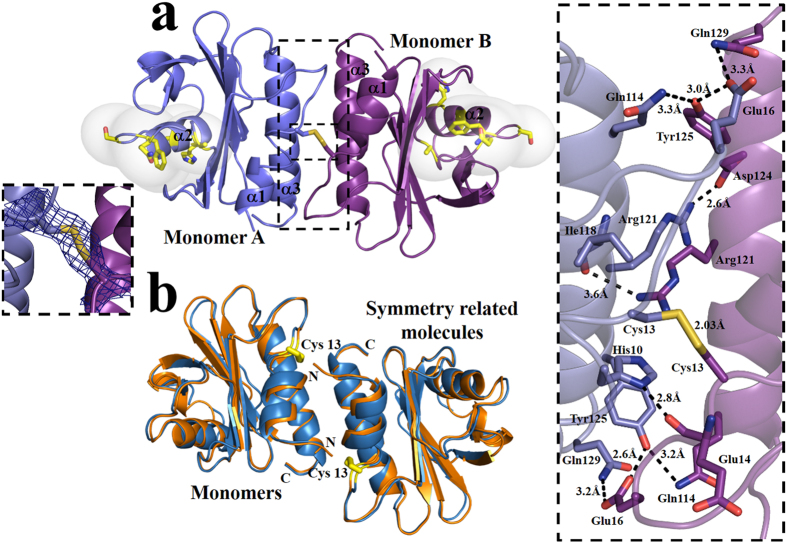
Interactions in the dimeric structures of profilin allergens. (**a**) Ribbon representation of the dimeric rHevb 8 in the asymmetric unit of the P3_2_ crystal, transparent surfaces show the residues (as sticks) that differ from rZea m 12. **Left insert,** 2Fo–Fc density map (σ = 1) of the disulfide bridge at the interface. **Right insert,** Close-up view of the dimer interface, the key residues involved in interactions are shown as sticks (PISA server). (**b**) 3D alignment of the monomers of rHev b 8 (blue) and rZea m 12 (orange) in the asymmetric units showing the symmetry related molecules that form monomer-monomer interfaces.

An analysis of the dimer interface of rHev b 8 (PDB code 5FEG) using the PISA (protein interfaces, surfaces, and assemblies) server showed an area of 674.3 Å^2^ in each monomer that includes the conserved α-helices 1 and 3. Figure 2a illustrates this interface that comprises a disulfide bridge between the conserved Cys13 of both monomers. The C2 symmetry of the dimer favors hydrogen bond interactions between some of the residues involved in binding poly-proline regions, such as Glu16, Gln114 and Tyr125 and Gln129, which are conserved residues in both allergens (Fig. 2 left insert). In addition, Arg121 in α-helix 3 of monomer A forms a salt bridge with Asp124 of monomer B in α-helix 3 (Fig. 2 right insert). Analyzing crystal packing, it was evident that α-helices 1 and 3 are important for the establishment of this interface, even in the monomeric crystal structures of rHev b 8 and rZea m 12 (5FDS and 5FEF respectively). In these two crystals, α-helices 1 and 3 are relatively closed to the symmetry-related molecule, albeit the disulfide bond is not established (Fig. 2b). It is also interesting to note that in both sequences, the residues that differ are exposed on the surface of the homodimer (Fig. 2a), which affect the electrostatic potential in each allergen (Fig. 1a). The assemblies searching for similar arrays in PISA showed that this interface is favorable for different yeast and plant profilin structures (PDB codes 1K0K, 1YPR, 3D9Y, 1G5U), even though the yeast profilins do not have the conserved Cys13 residue. Crystal packing analysis showed that the mean interface area for these structures, including 5FDS and 5FEF from this work, is approximately 562 Å^2^, which can be considered a medium size interface.

### Characterization of oligomeric profilins

**Figure 3:**
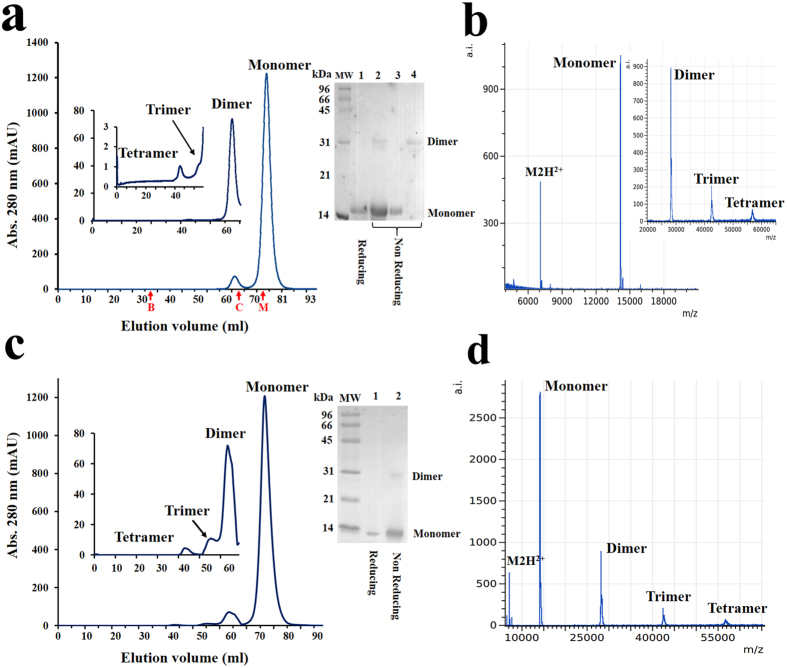
Characterization of profilin oligomers. (**a**) Size exclusion chromatogram of rHev b 8 applied to the column at a concentration 10 mg/mL. **Left inserts** show the fractions that eluted before the monomer and correspond to the dimer, trimer, and tetramer. Elution volumes for the controls are shown in red: M (Myoglobin), C (α-Chymotrypsin), B (BSA). **Right insert,** SDS-PAGE gel. Lane 1, rHev b 8 (10 mg/mL); Lane 2, the same sample in non-reducing conditions; Lanes 3 and 4, monomer and dimer after SEC under non-reducing conditions. **(b)** MALDI-TOF spectrum for rHev b 8, showing the peak corresponding to the monomer (m/z 14.1 kDa), the insert shows peaks corresponding to the dimer (28.3 kDa), trimer (42.4 kDa) and even the tetramer (56.5 kDa). (**c**) and (**d**) depict the same experiments for rZea m 12. For this protein lanes 1 and 2 in the SDS-PAGE show the sample before applied to the SEC column (10 mg/mL) under reducing and non-reducing conditions.

Our experimental results in solution suggested that both profilins oligomerize, usually as dimers. When the proteins purified by Ni-affinity chromatography were applied to a size exclusion chromatography (SEC) column using concentrations of 10 mg/mL, there was an increase of one fraction that corresponds to a profilin dimer (Fig. 3a). Low intensity peaks corresponding to the trimer, and tetramer were eluted first from the column (Fig. 3a left insert). SDS-PAGE of the monomeric and dimeric forms rHev b 8 before and after the SEC, under non-reducing and reducing conditions, is shown in Fig. 3a (right insert). It is evident that when the protein reaches a concentration of 10 mg/mL and under non-reducing conditions a homodimer covalently bound by a disulfide-bond was maintained. The mass spectra of these samples confirmed the presence of monomeric, dimeric, trimeric, and even tetrameric species (Fig. 3b). The SEC, mass spectrum and SDS-PAGE for rZea m 12 also confirmed the presence of oligomeric species (Fig. 3c,d).

### Generation of a high affinity mouse monoclonal IgE anti-profilin antibody (mAb 2F5)

**Figure 4:**
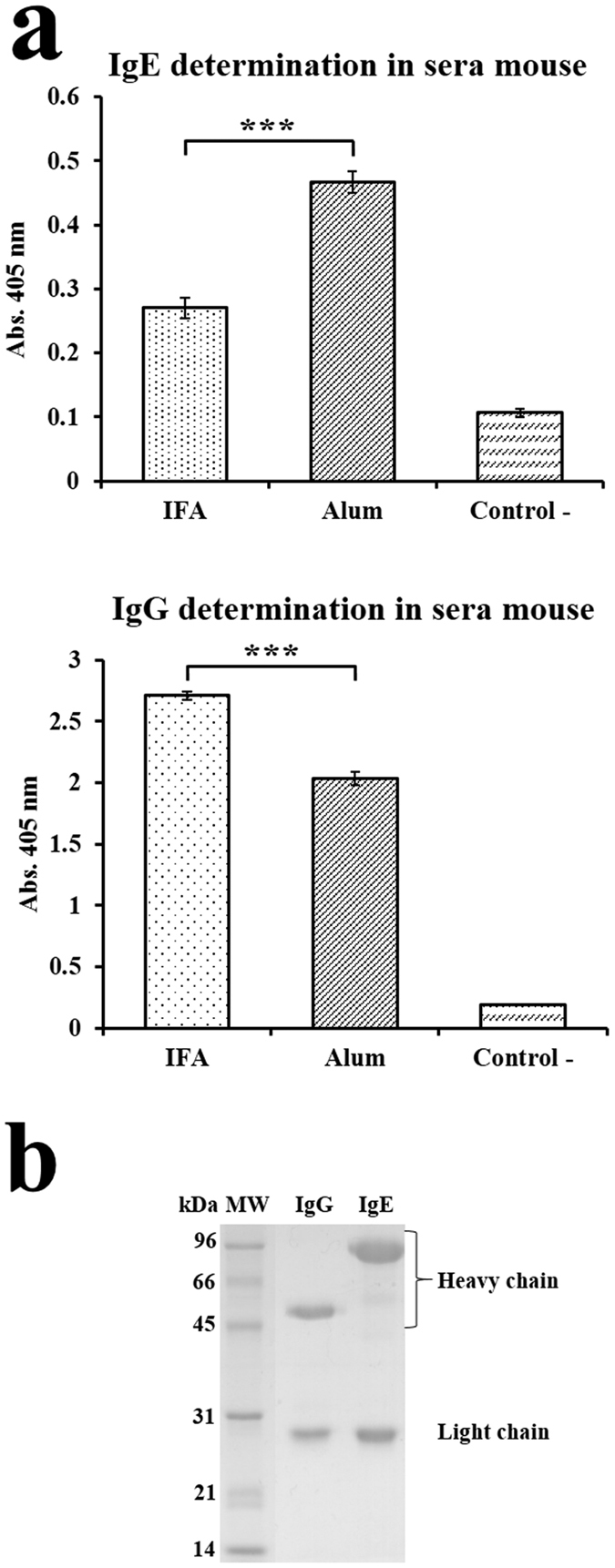
Determination of specific IgE and IgG mice serum antibodies at the end of the immunization protocol with rHev b 8. (**a**) ELISA experiments for class determination for the two adjuvants used (Incomplete Freund’s and alum). The negative control corresponds to the mouse without sensibilization (*** = *P* < 0.001). (**b**) SDS-PAGE of the IgE (mAb 2F5) and a IgG mouse purified by an affinity column with rHev b 8.

An interesting observation in this work was the response of the Balb/c mice upon immunization with rHev b 8 in the presence of either alum or IFA as an adjuvant. The results of the indirect ELISAs to measure the IgE and IgG levels in the sera of the immunized mice showed that higher levels of IgE were produced with alum than with IFA or no adjuvant (negative control). Conversely, when the immunization included IFA as the adjuvant, the generation of mouse IgG was predominant (Fig. 4a). The difference in the IgE response between the two adjuvants is significant with *P* < 0.001. Using the fusion of splenocytes from the mouse immunized with rHev b 8 adsorbed on alum, with the Ag8 cell line we obtained a hybridoma producing an IgEκ monoclonal antibody (mAb 2F5). SDS-PAGE of this IgE showed a band of ~75 kDa, which corresponds to the molecular mass of the epsilon heavy chain (Fig. 4b), whereas the gamma heavy chain of an anti-Hev b 8 murine monoclonal IgG1 is ~50 kDa.

**Figure 5:**
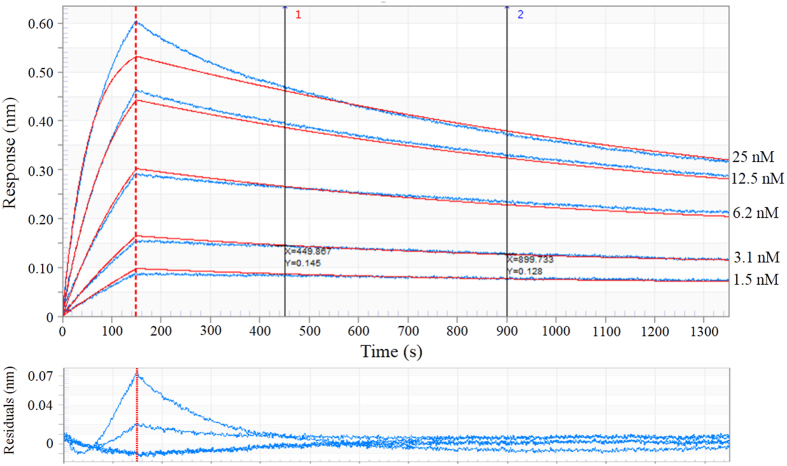
Biolayer interferometry sensorgram of rHev b 8 binding to mAb 2F5. Five different concentrations of mAb 2F5 were used. The red lines indicate the fits, whereas the sensorgram is in blue for each concentration. The residual of the fit is plotted below the sensorgram. The global quality average values are shown with the *R*^2^ (0.9945) and *X*^2^ (0.7173) considering a 2:1 model. All steps were performed at 25^o^C.

We used biolayer interferometry to obtain the kinetic association and dissociation constants (k_a_, k_d,_ and K_D_) for the interaction between rHev b 8 and mAb 2F5 (Fig. 5). A high correlation (*R*^*2*^ = 0.99) between the different curves demonstrated a fast association rate (150 seg; k_a_ = 3.66 × 10^−5^ s^−1^), and a slow dissociation rate (1200 seg; k_d_ = 4.94 × 10^−4^ s^−1^) when using an ionic strength of 0.75 M NaCl at 25 °C. These rate constants result in an equilibrium dissociation constant (K_D_) of 1.35 × 10^−9 ^M, which indicates a high affinity of the mAb 2F5 for rHev b 8.

### Differences in the recognition of rHev b 8 and rZea m 12 by mAb 2F5 and a pool of allergenic patients’ sera

**Figure 6:**
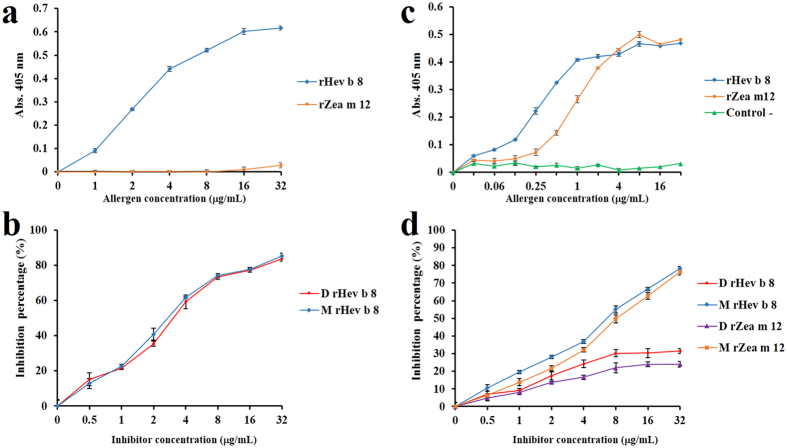
Immunological characterization of rHev b 8 and rZea m 12 allergens using mAb 2F5 and IgE from patients’ sera. (**a**) Recognition of the two allergens by mAb 2F5 using increasing concentrations of the allergens (*P* < 0.001). (**b**) Competitive ELISA experiment for mAb 2F5 binding to the monomer of rHevb 8 (coated in plate) in the presence of increasing concentrations of monomeric and dimeric rHev b 8 as competitor. (**c**) Recognition of rHev b 8 and rZea m 12 by a pool of patients’ sera (polyclonal IgEs). Control (non-allergic individuals) (*P* < 0.001). (**d**) Competitive ELISA experiments for human IgEs between monomers (coated in plate) and increasing concentration of monomeric and dimeric rHevb8 and rZea m 12, separately. However, there is a significant difference between monomer and dimers for both allergens (*P* < 0.001).

An indirect ELISA was used to compare the binding of mAb 2F5 and a pool of sera from profilin allergic individuals (n = 20) to rHev b 8 and rZea m 12, in their monomeric and dimeric forms. The assays showed that mAb 2F5 specifically recognizes monomeric rHev b 8, even at a low concentration (2 μg/mL) (Fig. 6a); conversely, mAb 2F5 showed no reactivity towards monomeric rZea m 12, suggesting that this IgE recognizes a conformational epitope on the surface of rHev b 8 that is not shared with rZea m 12. Competition assays with dimeric and monomeric rHev b 8 indicated there was no significant difference in their recognition by mAb 2F5 (Fig. 6b). When the pool of sera from profilin-allergic patients was used we found that both profilins were recognized to a similar extent, even though a lower concentration of rHev b 8 was required to obtain the maximum response (*P* = 0.001) (Fig. 6c). Competitive ELISAs using the IgEs from patients’ sera with both allergens, in their monomeric and dimeric forms, showed a similar recognition (Fig. 6d). However, between the monomeric and dimeric forms of both allergens we observed a significant difference in the IgE binding (*P* < 0.001), which suggests that the monomer has additional IgE epitopes in the region that establishes the homodimer interface. These results confirm the presence of a conformational epitope in the poly-proline binding region of profilins, as was proposed by Fedorov *et al*.^18^.

### IgE-mediated degranulation of RBL-2H3 cells induced by dimeric rHev b 8

**Figure 7:**
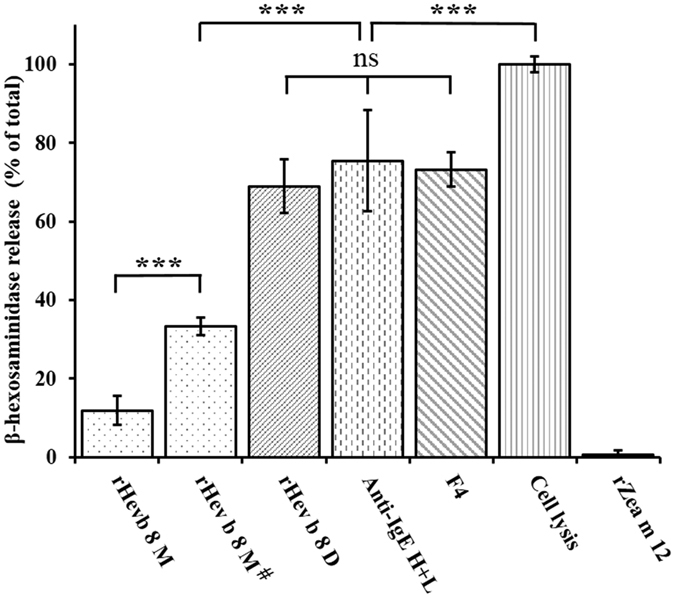
RBL-2H3 cell mediator release assay. Cell lysis represents 100% of the total content of β-hexosaminidase in the cells. Bars show the percentage of the enzyme induced by each stimulus. Direct (mAb F4 anti FcɛRI receptor) and Indirect (Anti-IgE H + L) controls were included. The negative control used is rZea m 12, which is not recognized by mAb 2F5. The first two bars correspond to monomeric rHev b 8 at two concentrations (130 nM y 260 nM^**#**^) and the third one corresponds to dimeric rHev b 8 (130 nM) (ns = not significant, *** = *P* < 0.001).

RBL-2H3 cells sensitized with mAb 2F5 degranulate upon stimulation with the monomeric and dimeric forms of rHev b 8 profilin (Fig. 7). This degranulation response, as measured by the release of β-hexosaminidase, was significant (~70%) when dimeric rHev b 8 (130 nM) was used, and of a similar magnitude to the response obtained with either IgE complexed with an anti-IgE secondary antibody (133.3 nM), or the anti-FcεRI murine IgG (F4) antibody (66.7 nM) (positive controls). When the cells were stimulated with monomeric rHev b 8 using the same and two fold the concentration of the dimer (130 and 260 nM, respectively) the release of β-hexosaminidase reached near half of the homodimer, and the positive controls responses *P* < 0.001 (Fig. 7). The negative control was monomeric rZea m 12 (260 nM), which was not recognized by mAb 2F5, as established by indirect ELISA (Fig. 6a).

## Discussion

There has recently been an increased interest in analyzing allergen structures in their oligomeric forms and how oligomerization affects their recognition by different antibodies^2^,^19^. In this work, we focus on the study of two plant pan-allergens, profilins from the rubber tree (rHev b 8) and maize (rZea m 12), in their monomeric and dimeric forms. Using SEC we isolated dimeric species of both allergens and observed the presence of other oligomeric species, confirmed by mass spectrometry (Fig. 3). Analyzing the different SEC fractions by SDS-PAGE in non-reducing conditions we observed mainly monomers and dimers, whereas reducing conditions cause the disappearance of the 28-kDa band. These results suggest that both profilins self-assemble to form a covalent bond, but also noncovalent interactions are established that explain the presence of transient oligomers when the protein concentration is high and protein-protein interactions are favored. Likewise, the presence of endogenous profilin oligomers in yeast, human and plants has already been demonstrated^20^.

In this study, we determined the tridimensional structure of a dimeric rHev b 8 stabilized by a disulfide bridge and compared it with the structures of the monomeric forms of the same allergen and that of rZea m 12. At the structural level, the sequence alignment and 3D structures of both allergens (Fig. 1) revealed that the secondary structure elements and protein folding were maintained, with slight differences between the monomers. Interestingly, the dimeric structure of rHev b 8 displays a medium size interface stabilized through a disulfide bond that is formed between the two Cys13 residues of each monomer, which is conserved in all plant profilins^21^. In addition, there are several hydrophobic and polar interactions between residues of α-helix 3 at the C-terminus and α-helix 1 at the N-terminal region (Fig. 2a right insert). Even though most of the profilin structures reported in the PDB show one protein chain in the asymmetric unit, when we analyzed the crystal contacts we found that the two helices at the N- and C-terminal regions are involved in a similar interface. For example, the profilins from yeast do not have Cys 13, but crystal contacts with symmetry related molecules revealed the presence of transient dimers^19^ with an interface that exhibits similar non-covalent interactions involving α-helices 1 and 3. From a functional point of view, the hydrophobic N- and C-terminal regions of the profilin structures are very important for their interaction with proline-rich proteins, such as kinases^22^,^23^. This region is blocked when the dimer is formed; therefore, interactions with kinases could be limited^24^. According to Aparicio-Fabre R, *et al*.^25^ the interaction of profilin with a phosphoinositide 3-kinase could affect several protein-protein interactions, which are important to regulate diverse cellular functions, including protein activity subcellular localization and association with proteins involved in the regulation of actin dynamics and endocytosis, among others. As observed in our dimer structure the interface blocks the kinase recognition region; therefore, we can suggest that the dimerization process might then have an auto-regulatory function for the members of the profilin family. In general, the most important structural differences between rHev b 8 and rZea m 12 are the contributions of some side-chains that alter the surface charge, essentially at the loop that has been considered important for antibody recognition (Fig. 1a)^26^,^27^,^28^ and it is exposed on the surface of the dimer structure (Fig. 1b).

To gain insight into the allergen-antibody interactions and how these might affect hypersensitivity reactions, we produced a murine IgE monoclonal antibody (mAb 2F5) that specifically recognizes the allergen rHev b 8. There are only a few studies in the literature that describe human and murine IgE antibodies produced by various techniques. The first reported monoclonal IgE derived from a hybridroma was an antibody anti-2,4-dinitrophenyl, which exhibited a K_D_ of 1.4 × 10^−8 ^M^29^. Two IgE monoclonal antibodies raised against different allergens have also been reported, the anti-Bos d 5 (β-lactoglobulin)^6^ and an anti-Phl p 2 (grass pollen allergen)^30^, with K_D_s of 1.4 × 10^−9 ^M and 1.1 × 10^−10 ^M, respectively. In our study, we used biolayer interferometry to characterize the kinetic binding of mAb 2F5 to rHev b 8, which produced a high apparent affinity constant (K_D_ = 1.3 × 10^−9 ^M) that is comparable to those reported previously for allergen-IgE complexes. However, in this study it was necessary to increase the ionic strength in order to measure the dissociation rate, suggesting that the affinity may be even higher at physiological ionic strengths.

From the immunological standpoint, it is relevant to note that mAb 2F5 only recognized rHev b 8, but not rZea m 12 (Fig. 6a), even though the sequence identity is 79.4% and the folding is very similar (rmsd of 0.27 Å for Cα). The main differences were observed in a flexible loop that connects the β-strand 2 with α-helix 2 (Fig. 1a), where the charge distribution could affect the IgE binding. In addition, the α-helix 2 in both profilins has several different side chains, which could affect interactions with the antibody mAb 2F5, corroborating the predictions by López-Torrejón *et al*.^27^. Interestingly, the flexible loop and α-helix 2 do not participate in the establishment of the rHev b 8 dimer interface (Fig. 2a) and the competitive ELISAs between monomeric and dimeric rHev b 8 (Fig. 6b) confirmed that this region is exposed for interaction with mAb 2F5. Nonetheless, when we evaluated the recognition by IgE in patients’ sera (polyclonal response), we demonstrated that both allergens in their monomeric and dimeric forms showed cross-reactivity (Fig. 6c,d), which could be explained by the presence of conserved regions related to their function (Fig. 1b,c) that have been reported as IgE epitopes^18^,^21^,^31^,^32^,^33^. Moreover, we confirmed that profilin monomers are multivalent, since they were recognized by polyclonal IgEs in competition assays with the dimeric forms of both allergens (Fig. 6d), which could mask some IgE epitopes in their interface; however, maintained other regions exposed for the recognition by antibodies.

We also analyzed the ability of mAb 2F5 to release allergic mediators from RBL-2H3 cells in response to monomeric and dimeric rHev b 8. Notably, we observed a significant degranulation response to dimeric rHev b 8, equivalent to the release induced by the positive controls (mAb F4 and anti-IgE) (Fig. 7). These results demonstrate that dimeric rHev b 8 efficiently stimulate the cross-linking of pre-bound mAb 2F5 to FcεRI receptors, activating degranulation. We also determined cell degranulation activity in the presence of the profilin monomer, at two different concentrations, which could be the result of a local increased protein concentration and explained by the colocalization phenomena. While it is true that only a minor fraction of Hev b 8 in solution forms dimers, it is very plausible that by binding to the IgE bound to the FcɛRI on the cell surface, the Hev b 8 concentration increases in the immediate vicinity of the membrane, facilitating the formation of “transient dimers”, that not necessarily involves the establishment of a disulfide bridge, as has been observed in the crystal contacts of other profilin structures including our monomeric structures (rHev b 8 and rZea m 12)(Fig. 2b). Moreover, because of the very high affinity of mAb 2F5 for Hev b 8, two monomers bound to different IgE-FcɛRI complexes, would be able to form dimers at a much higher rate than two monomers in solution, as their being bound to the cell surface would dramatically increase their concentration and reduce their motion to the lateral diffusion of the complexes on the cell membrane. In general, it has been established that protein concentration within cells is in the nanomolar to micromolar range; however, colocalization by nonspecific interactions may locally increase this concentration up to 1 mM^34^. Profilin is an abundant protein in pollen with a cellular concentration that is nearly equimolar to actin in some species^22^. Therefore, it is probable that profilins oligomerize *in vivo* similarly to what we observed in our solution experiments. This suggests that the response mediated by IgE antibodies using *in vitro* tests is established through the molecular recognition of profilin monomers or oligomers and the affinity and specificity that contribute to the allosteric properties of these antibodies when they are bound to FcεRI receptors.

Recent studies have suggested the importance of allergen dimerization in their recognition by IgE bound to specific receptors; thus, we produced, stabilized, and structurally characterized a profilin dimer, and determined the consequences of oligomerization regarding its recognition by murine monoclonal and human polyclonal IgE antibodies. Based on the results we suggest that differences in the immunological response among profilins from different sources are due to their multivalent characteristics that can be enhanced by the existence of dimeric species. Besides, the information about the localization of specific IgE epitopes on profilin and how these are exposed on the dimers surfaces is important for the determination of cross-reactivity among the members of this family of panallergens. Our data indicate the relevance of slight changes in the sequence of plant profilins, mainly in non-conserved regions, which significantly affect their recognition by IgE antibodies. However, we also stress that the conserved regions related to the biological function of these proteins are also immunogenic epitopes that can be responsible for allergic cross-reactions in the pollen-latex-food syndrome, as we could determine with the profilins from the rubber tree and maize, thus contributing to the design of effective diagnostic tools and immunotherapy strategies considering the important increase of allergies in recent years.

## Materials and Methods

### Cloning, expression, and the purification of profilins from *Hevea brasiliensis* (rHev b 8) and *Zea mays* (rZea m 12)

Total RNA was extracted from the young leaves of the rubber tree *H. brasiliensis*, clone RRIM600, as previously reported^35^ and from *Z. mays* leaves with the TRizol Plus RNA Purification Kit (Invitrogen, Carlsbad, CA), as described by the manufacturer. The cDNA was synthesized from total RNA using the cDNA synthesis kit (New England Biolabs, MA, USA) and amplified with oligo-dT primers. PCR was carried out using specific primers (Supplementary Table S1) for the isoform Hev b 8.0102 (named here rHev b 8), which were designed to amplify the gene according to the sequence reported by Rihs *et al*.^36^ (GenBanK accession number CAB51914.1). This isoform is different to the one previously reported in the PDB (Hev b 8.0203, PDB code 2G5U). Primers for Zea m 12.0105 (rZea m 12) amplification (Supplementary Table S1) were designed using the sequence reported by Kovar *et al*.^37^ (GenBank accession number AAG35601.1). The PCR product was ligated into the pET28c vector (Novagen EMD Biosciences, WI, USA) using T4 DNA ligase and then transformed into *E. coli* DH5α and sequenced (Laragen, CA, USA).

For the expression of proteins the pET28c-rHev b 8 and pET28c-rZea m 12 vectors were cloned separately in *E. coli* Rosetta (DE3) cells. These were grown in Luria broth (LB) medium supplemented with 30 μg/mL kanamycin at 37 °C with shaking. When the optical density at 600 nm reached 0.6, the expression of both proteins was induced with Isopropyl-thio-β-D-1-galactopyranoside 0.5 mM and growth was continued for 6 h at 30 °C. Cells were harvested by centrifugation (15,300 *g*, 4 °C, 20 min). SDS-PAGE analysis on a 15% gel was performed to estimate the expression levels of both proteins. The cell pellets were resuspended in a lysis buffer (50 mM Tris pH 8.0, 0.3 M NaCl, 1 mM PMSF) and then subjected to sonication (60 cycles in 10 s intervals, with a stop time of 30 s to complete 10 min, 4 °C). The lysate was clarified by centrifugation (26,000 g, 4 °C, 30 min); the supernatant was filtered and loaded into a 5 ml Ni-NTA affinity column. This was equilibrated with 50 mM Tris-HCl pH 7.5, 20 mM imidazole buffer and bound proteins were eluted with an imidazole gradient. rHev b 8 and rZea m 12 proteins were dialyzed against 50mM Tris-HCl pH 7.5 buffer. Protein concentration was estimated by Abs_280_, using a molar extinction coefficient of 19940 M^−1^cm^−1^ for rHev b 8 and 16960 M^−1^cm^−1^ for rZea m 12. The allergen rHev b 8 was separated from the His_6_tag by incubating with enterokinase (Invitrogen, CA, USA) overnight at 37 °C, according to the manufacturer’s instructions. The final purification step of the recombinant rHev b 8 was performed using anion exchange chromatography with a mono Q HR 5/5 column in an ÄKTA FPLC (Amersham Biosciences, Uppsala, Sweden). For the rZea m 12 construction, the Histag was cleaved overnight at 4 °C using a recombinant tobacco virus protease (TEV) in 50 mM Tris-HCl pH 7.5, 0.5 mM EDTA and 1 mM DTT buffer. An additional Gly residue was left at the N-terminus after digestion with TEV in rZea m 12. The protein mixture was dialyzed against 50 mM Tris-HCl pH 7.5 buffer and then loaded onto a 5 mL Ni-NTA affinity column equilibrated with the same buffer. The purified rZea m 12 did not bind to the column and was eluted. The pure allergens rHev b 8 and rZea m 12 were dialyzed against 50 mM Tris-HCl pH 7.5 and store at −20 °C.

### Isolation and characterization of rHev b 8 and rZea m 12 oligomers

To separate oligomers of the two purified profilin samples we used a HiLoad 16/60 Superdex^TM^ 75 size exclusion chromatography (SEC) column in an ÄKTA FPLC system (Amersham Pharmacia Biotech, AB, Sweden). After equilibration of the column with 50 mM Tris-HCl, pH 7.5, protein samples incubated for 1 week at 4 °C at two different concentrations (5 and 10 mg/mL) were applied. The column was calibrated using the molecular weight standards Myoglobin (M_r_ 16.95 kDa), α-chymotrypsin A (M_r_ 25 kDa), and bovine serum albumin BSA (M_r_ 66.43 kDa).

Matrix-assisted laser desorption/ionization-Time of flight (MALDI-TOF) Mass Spectrometry was used for determining molecular masses of the recombinant proteins using a Microflex Bruker spectrometer. One μl of each recombinant protein (10 mg/mL) was used for the analysis. Standards for mass calibration were myoglobin (M_r_ 16.95 kDa); cytochrome C (M_r_ 12.38 kDa); and BSA (M_r_ 66.43 kDa). The matrix used was a saturated solution of sinapinic acid in 40% (v/v) aqueous acetonitrile, 0.1% (v/v) trifluoroacetic acid. Samples were analyzed with the software Flex analysis 3.0^TM^ (Bruker).

### Crystallization

The proteins rHev b 8 and rZea m 12 were dialyzed against a 0.05 M Tris HCl pH 7.5 buffer, with and without 1 mM DTT, using an ultrafiltration membrane with a MWCO of 5 kD and concentrated to 5 mg/mL and 10 mg/mL. The hanging-drop vapor-diffusion method and 24-well Linbro plates were used in conjunction with solutions of the Hampton Research Crystal Screens I and II (Hampton Research, CA, USA) to search for suitable crystallization conditions. The final optimized crystallization condition for both allergens was 0.1 M Tris HCl (pH 9.0) and 1.6 M ammonium sulfate with a protein concentration of 5 mg/mL. These protein crystals belong to the space group P4_3_2_1_2. Only one crystal grew for the allergen rHev b 8 using a protein concentration of 10 mg/mL that belonged to the space group P3_2._

### Data collection, structure determination and refinement

Diffraction data for the rHev b 8 trigonal (P3_2_) crystal was collected using a rotating-anode generator (Cu κα, λ = 1.5416 Å) with a Rigaku R-AXIS IIC image-plate detector (Rigaku, TX, USA). This crystal contained two molecules in the asymmetric unit. For the rHev b 8 and rZea m 12 tetragonal crystals (P4_3_2_1_2) that contained a monomer in the asymmetric unit, diffraction data were collected on a Rigaku MicroMax-007 HF rotating anode (Rigaku, TX, USA) at 103°K with an oscillation of 0.5°/frame on an R-AXIS IV++ image plate detector. Mother liquor including 30% glycerol was used as cryoprotectant in all cases. All data sets were indexed and integrated using XDS^38^, and reflections merged and scaled with Scala in the CCP4 suite^39^. The correct space groups were determined in all cases with Pointless^40^. The 3D structures were solved using molecular replacement techniques with the program Phaser in the Phenix suite^41^. For the monomeric rHev b 8 (P4_3_2_1_2) we used as a template the structure of profilin I from *Arabidopsis thaliana* (PDB code 3NUL), and the final refined model of this crystal (PDB code 5FDS) was used as template for determining the structures of the dimeric rHev b 8 and of rZea m 12. In all cases, map inspection and subsequent model building were performed with COOT^42^, and the resulting models were refined with phenix-refine^43^. The P3_2_ crystal exhibited merohedral twinning, but refinement was more stable when the twinning operator was included. The final structures satisfied the MolProbity criteria at the corresponding resolutions^44^.

### Structures analysis

The three final models were drawn and compared using PYMOL v.1.7.x (Schrödinger)^45^, and the electrostatic surface potentials were obtained at pH 7.0 with the Adaptive Poisson-Boltzmann Solver (APBS)^46^ software package. Sequence alignment was performed using the PRALINE server^47^. Analysis of the dimer interface and the search of similar assemblies for the comparison with other profilins were performed using the PISA web server (European Bioinformatics Institute)^48^.

### Ethics Statements

All animal handling and experimental procedures were performed in accordance with the recommendations in the Guide for Care and Use of Laboratory Animals and the Mexican Official Norm NOM: 062-ZOO-1999. The protocol was approved by the “Comité Institucional para el Cuidado y Uso de animales de laboratorio (CICUAL)” of the Instituto de Investigaciones Biomédicas, Universidad Nacional Autónoma de México (Permission number CICUAL Protocol ID 98).

Human sera from control and profilin-allergic individuals with medical records of pollen, fruits and latex allergies were provided by the Instituto Nacional de Enfermedades Respiratorias “Ismael Cosio Villegas” (INER), México. The institutional Ethical Committee approved the study and all subject provided an informed consent. Experimental protocols were performed in accordance with the approved guidelines of the Institution.

### Murine monoclonal antibody production and purification

Female Balb/c mice (eight weeks old) were subcutaneously immunized with 50 μg of rHev b 8 either adsorbed in 200 μL of alum (Al(OH)_3_) or emulsified with incomplete Freund’s adjuvant (IFA), every two weeks during 2 months. Two additional intraperitoneal immunizations with 25 μg of allergen in PBS were applied, 3 and 4 days prior to the fusion process. Sera of the immunized mice were tested with an indirect ELISA using a rat monoclonal [23G3] anti-mouse IgE (epsilon chain-specific) secondary antibody labeled with Horseradish peroxidase (HRP) (Abcam Inc. Cambridge, U.K.), and also using a goat anti-Mouse IgG H+L secondary antibody HRP (Pierce Thermo-scientific, IL, USA). The mouse with a positive IgE response to the allergen was selected for cell fusion.

The hybridoma cells were derived by somatic cell hybridization of P3 × 63Ag88.653 mouse myeloma cell line to spleen cells from the selected immunized mouse at a ratio of 1:5 in the presence of 41% polyethylene glycol (PEG) 1550. The fusion cells were plated on 96-well flat-bottom plates at a density of 1 × 10^5^ cells in RPMI-1640 medium and were selected by Hypoxanthine-Aminopterin-Thymidine (HAT) medium as previously described^49^. Ten days after the fusion, the supernatants of hybridoma cells were tested for the presence of IgE or IgG antibodies that recognized rHev b 8 bound to an ELISA plate. We isolated one clone secreting IgE anti-profilin antibody (mAb 2F5) and several clones secreting IgG antibodies. Hybridomas secreting rHev b 8-specific IgE or IgG antibodies were cloned by limiting dilution. The isotype of the monoclonal antibodies was determined using the mouse immunoglobulin isotyping kit (Invitrogen, CA, USA).

For monoclonal antibody production, hybridoma cells were grown in RMPI-1640 media supplemented with L-glutamine, and 3% of fetal bovine sera (FBS). The mAb 2F5 IgE was purified from the centrifuged and filtered (0.22 μm membrane) supernatant by affinity chromatography with rHev b 8-Affi-gel 15 column (Biorad, CA, USA) equilibrated with PBS buffer. The monoclonal IgE antibody was eluted from the column using 0.2 M Glycine-HCl pH 2.8 and received in 2 M Tris-HCl pH 8.0 for neutralization of the pH. The purified IgE was dialyzed against PBS, concentrated to 1 mg/mL and stored at −20 °C. The concentration was determined using an extinction coefficient^29^ (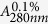) of 1.62 mL mg^−1^ cm^−1^ for an IgE murine anti-DNP. All procedures were performed at 4 °C.

### Binding kinetics using biolayer interferometry (BLI)

To determine the affinity of the mAb 2F5 IgE for the allergen rHev b 8, a kinetic titration was performed at 25 °C using streptavidin biosensors in the HTX Red384 Octec systems (FortéBio Inc., CA, USA). Biotin was coupled to rHev b 8 using amino reactive-free conditions with 0.1 M 3-morpholinopropane-1-sulfonic acid, pH 7.2, as the coupling buffer with excess reactive esters blocked with ethanolamine. The first step consisted of establishing a zero baseline using the BLI buffer [phosphate buffered saline (PBS), pH 7, 0.05% Tween 20, 0.5% bovine serum albumin (BSA)] for 60 s. The second step entailed loading biotinylated rHev b 8 and capturing it for 300 s to activate the sensor, after which a new baseline was obtained. To determine the association constant (k_a_), five different concentrations of purified mAb 2F5 were bound to the immobilized rHev b 8 for 150 s. Finally, the dissociation constant (k_d_) of the antibody-allergen complex was determined over 1200 s using the BLI buffer with different concentrations of NaCl. Immobilized BSA was used as a negative control, and a new biosensor tip was used for each run. The Octec data were processed with the FortéBio data acquisition software using a global fitting approach.

### Recognition of rHev b 8 and rZea m 12 by mAb 2F5 and human IgE using indirect ELISA

Enzyme-linked immunosorbent assays (ELISA) were performed in 96-well microplates. The wells were coated with 100 μL of each allergen in PBS pH 7.4 (8 μg/mL) for 2 hours at 37 °C. After washing with PBS/0.1% Tween 20, the plates were blocked with PBS/1% BSA for 2 hours at 37 °C. One hundred microliters of sera from profilin allergic individuals diluted 1:10, sera from immunized mice diluted at either 1:10 (IgE determination) or 1:100 (IgG determination), and mAb 2F5 IgE (2 μg/mL) were added to their corresponding wells. The plates were then incubated for 3 hours at 37 °C. Depending on the antibody being tested, the subsequent steps included the addition of anti-human IgE-horseradish peroxidase (HRP; 1:1000), anti-mouse IgE (epsilon specific chain)-HRP (1:1000) or Anti IgG mouse H&L-HRP (1:5000) before incubating for 1 hour at 37 °C. The peroxidase reaction was developed using 2, 2’-azino-di-(3-ethyl-benzthiazoline-6-sulphonic acid) and was read at 405 nm in a microplate reader. Each absorbance value was calculated from the mean of three independent determinations and standard deviation was determined.

### Recognition of rHev b 8 and rZea m 12 and their dimeric forms by competitive ELISA

We used a pool of sera from twenty patients allergic to profilin and the purified mAb 2F5, separately. Microwells were coated with monomeric rHev b 8 or rZea m 12 (8 μg/mL) and blocked with 1.0% BSA in PBS. In separate tubes the pool of patients’ sera (diluted 1:10 in PBS/0.1% BSA/0.05%Tween 20) and the mAb 2F5 (0.5 μg/mL) were incubated with serial dilutions of the competing dimeric rHev b 8 or rZea m 12 at 37 °C for 1 h, in both cases BSA was used as negative control. Then, 50 μl of the incubated samples were added to the plates and incubated to 37 °C for 1 h. The rest of the procedure was performed as described above for the indirect ELISA.

### Degranulation of basophils (RBL-2H3) determined by β-hexosaminidase release

The enzymatic activity of β-hexosaminidase that is contained in the cell’s granules was monitored. The cells were seeded in 96-well tissue-culture plates at 1 × 10^5^ cells in 100 μL of Eagle’s minimal essential medium with Earle’s salt (MEM) supplemented with 10% fetal bovine serum, and incubated at 37 °C in 5% CO_2_ for 5 h. Then sensitization of the cells with IgE was performed by the addition of 50 μL of the mAb 2F5 (2 μg/mL) in MEM per/well and the plate was incubated for 12 hours at 37 °C in 5% CO_2_. The monolayers were washed three times with Tyrode’s buffer to eliminate the mAb 2F5 excess. Allergens were added to the washed monolayers at different concentration to get the maximum degranulation. As positive controls for the cell’s FcεRI-mediated degranulation, we stimulated them with either a mouse monoclonal anti-FcεRI (F4) antibody^50^ (in non-sensitized cells) or with a Goat anti-mouse IgE H&L when the mAb 2F5 was already bound to the cells. Total β-hexoaminidase activity was determined in wells where the cells were treated with lysis buffer (Tris-HCl 50 mM, 150 mM NaCl, and 1% Triton X-100 to pH 7.5 with cocktail of proteases inhibitors). The rZea m 12 was used as a negative control. After the treatments indicated for each experiment, secretion was allowed to proceed for 30 min at 37 °C. From each well, three aliquots of 25 μL were transferred to a separate plate and 50 μL of the substrate solution (1.3 mg/mL p-nitrophenyl-N-acetyl-β-D-glucosamine in 0.1 M citrate, pH 4.5) was added before incubating the plates for 90 min at 37 °C. The reaction was stopped with 150 μL of 0.2 M glycine (pH 10.4), after which the absorbance was measured at 405 nm. The results are expressed as a percentage of the total β-hexosaminidase activity present in the cells. The spontaneous release observed in the absence of stimulus was subtracted from each experimental value.

### Statistical analysis of experimental data

The statistical analysis of the ELISA and degranulation data was performed using the one way-analysis of variance (ANOVA), followed by an all-pairwise multiple comparison procedures (Holm- Šídák method). A *P*-value < 0.05 was considered statistically significant. All summary statistics and analyses were performed using the software SigmaPlot 12.0 (www.sigmaplot.com).

## Additional Information

**Accession codes:** Coordinates and structure factors have been deposited in the Protein Data Bank (PDB) with accession codes: 5FDS, 5FEF, and 5FEG.

**How to cite this article**: Mares-Mejía, I. *et al*. Structural insights into the IgE mediated responses induced by the allergens Hev b 8 and Zea m 12 in their dimeric forms. *Sci. Rep.* **6**, 32552; doi: 10.1038/srep32552 (2016).

## Supplementary Material

Supplementary Information

## Figures and Tables

**Table 1:** Data collection and refinement statistics.

	Dimeric rHev b 8	Monomeric rHev b 8	Monomeric rZea m 12
**PDB code**	5FEG	5FDS	5FEF
**Data collection**			
Space group	P3_2_	P4_3_2_1_2	P4_3_2_1_2
Cell dimensions			
*a*, *b*, *c* (Å)	58.56, 58.56, 87.34	59.04, 59.04, 132.89	58.53, 58.53, 135.20
*γ* (°)	120		
Resolution (Å)	33.09-2.8 (2.95-2.8)*	33.22-1.9 (2.0-1.9)*	44.25-2.2 (2.32-2.2)*
No. unique reflections	8077 (1168)	19125 (2665)	12489 (1745)
*R*_merge_†(%)	6.8 (47.5)	5.1 (44.7)	5 (48)
*I*/σ*I*	8.9 (2.1)	18.9 (3.7)	17.2 (3.2)
Completeness (%)	98.1 (97.6)	99.2 (98.2)	99.2 (98.2)
Redundancy	2.6 (2.5)	4.9 (4.9)	4.5 (4.6)
B factor from Wilson plot (Å^2^)	58.1	25.38	42.4
**Refinement**			
*R*_work_/*R*_free_‡	20.78/24.95	17.3/19.7	21.8/24.5
No. atoms	1914	1187	1073
Protein	1914	1045	1011
Water	0	131	32
Ligand molecules	0	11	30
RMS-bonds (Å)	0.009	0.007	0.008
RMS-angles (°)	1.26	1.06	1.24
Mean *B*-factor (Å^2^)	51.98	27.74	48.04
Protein	51.98	26.29	47.13
Ligand	0	65.85	78.73
Water	0	36.11	48.11
Ramachandran favored/outliers (%)	87/2.3	97/0	98/0

^*^Highest resolution shell is shown in parenthesis.

^†^R_merge_ = Σ_hkl_Σ_i_ |I_i_(hkl) − <I(hkl)>|/Σ_hkl_Σ_i_ I_i_(hkl).

^‡^R_work_ = Σhkl │|Fobs| − |Fcalc|│/Σhkl |Fobs|. Rfree is the corresponding R-factor value for a randomly chosen 5% of the reflections excluded from the refinement.
