# Supplementary material for: Structural insights into the IgE mediated responses induced by the allergens Hev b 8 and Zea m 12 in their dimeric forms
Source: Sci Rep. 2016 Sep 2;6:32552. doi: 10.1038/srep32552 (PMC5009318; doi:10.1038/srep32552)
Supplement: Supplementary Information [file srep32552-s1.pdf]

Supplementary information

Structural insights into the IgE mediated responses induced by the allergens Hev b 8 and Zea m 12 in their dimeric forms

Israel Mares-Mejía[1], Siseth Martínez-Caballero[1], Claudia Garay-Canales[2], Patricia Cano-Sánchez[1], Alfredo Torres-Larios[3], Samuel Lara-González[4], Enrique Ortega[2], & Adela Rodríguez-Romero[1*]

[1]Instituto de Química, [2]Instituto de Investigaciones Biomédicas, [3]Instituto de Fisiología Celular, Universidad Nacional Autónoma de México, Circuito Exterior, Cd. Universitaria, Coyoacán, Ciudad de México, 04510. [4]Instituto Potosino de Investigación Científica y Tecnológica, Camino a la Presa San José 2055, Col. Lomas 4a. Sección, San Luis Potosí, México, 78216.

[*]**Correspondence**: Adela Rodríguez-Romero, Instituto de Química, Universidad Nacional Autónoma de México, Circuito Exterior, Cd. Universitaria, Coyoacán, Ciudad de México, 04510. Telephone: 5255-56224568, Fax: 5255-52162217, E-mail: adela@unam.mx

Table S1. Primers design.

**rHev b 8 + Enterokinase site**	**rZea m 12 + TEV site**
Forward: 5`-CGC*GGATCC*GACGACGACGACAAGATGACGTGGCAGGCGTACGTAGATGATCAT-3`<br><br>Site: *BamH I*	*Forward: 5'-TAC*AGATCT*GAAAACTTGTATTTCCAGGGCATGTCGTGGCAGGCGTACGTCGATGACCACCTGCTGTGCGACATC-3'<br><br>Site: *BglII*
Reverse: 5`-GGT*AAGCTT*TTAATAGCCCTGATCAATGAGGTA-3`<br><br>Site: *Hind III*	Reverse: 5'-GGT*AAGCTT*TTAGAAGCCCTGTTCGATCAGGTA-3'<br><br>Site: *Hind III*

*The TEV Site in rZea m 12 leaves a glycine residue in the N-terminus
